# Image Corruption Detection in Diffusion Tensor Imaging for Post-Processing and Real-Time Monitoring

**DOI:** 10.1371/journal.pone.0049764

**Published:** 2013-10-25

**Authors:** Yue Li, Steven M. Shea, Christine H. Lorenz, Hangyi Jiang, Ming-Chung Chou, Susumu Mori

**Affiliations:** 1 The Russell H. Morgan Department of Radiology and Radiological Science, The Johns Hopkins University School of Medicine, Baltimore, Maryland, United States of America; 2 Department of Biomedical Engineering, The Johns Hopkins University School of Medicine, Baltimore, Maryland, United States of America; 3 Center for Applied Medical Imaging, Siemens Corporate Research and Technology, Baltimore, Maryland, United States of America; 4 Department of Medical Imaging and Radiological Sciences, Kaohsiung Medical University, Kaohsiung, Taiwan, ROC; University of Manchester, United Kingdom

## Abstract

Due to the high sensitivity of diffusion tensor imaging (DTI) to physiological motion, clinical DTI scans often suffer a significant amount of artifacts. Tensor-fitting-based, post-processing outlier rejection is often used to reduce the influence of motion artifacts. Although it is an effective approach, when there are multiple corrupted data, this method may no longer correctly identify and reject the corrupted data. In this paper, we introduce a new criterion called “corrected Inter-Slice Intensity Discontinuity” (cISID) to detect motion-induced artifacts. We compared the performance of algorithms using cISID and other existing methods with regard to artifact detection. The experimental results show that the integration of cISID into fitting-based methods significantly improves the retrospective detection performance at post-processing analysis. The performance of the cISID criterion, if used alone, was inferior to the fitting-based methods, but cISID could effectively identify severely corrupted images with a rapid calculation time. In the second part of this paper, an outlier rejection scheme was implemented on a scanner for real-time monitoring of image quality and reacquisition of the corrupted data. The real-time monitoring, based on cISID and followed by post-processing, fitting-based outlier rejection, could provide a robust environment for routine DTI studies.

## Introduction

Diffusion tensor imaging (DTI) is an MRI technique that measures the anisotropy of water incoherent motion, which can generate unique image contrasts to display the brain white matter [1], [2]. In DTI, a diffusion tensor is typically estimated by a least squares error fit of the intensity at each pixel of diffusion-weighted images (DWIs) and non-diffusion-weighted (b0) images. Sensitive to molecular motion on the order of 10 µm, DWIs often suffer from a large amount of signal loss (corruption). An image slice that is acquired during bulk motion could lead to complete signal loss of the entire slice. Even without bulk motion, sub-pixel elastic brain motion caused by cardiac pulsation is known to cause regional signal loss [3]–[8]. The image corruption leads to errors in the subsequent tensor estimation. Although the mis-registration can be lessened by post-processing image alignment, for corrupted images, the only available solution is to discard the affected image slice (slice rejection) or pixels (pixel rejection).

To reduce the impact of the image corruption, several artifact detection methods have been [9]–[11] proposed. One of the most widely used methods is the robust estimator approach [10], [11], in which a tensor is first estimated with all data points using a robust estimator, and data points with large fitting errors (differences between calculated and measured diffusion-weighted values) are rejected. Most of these methods use an iterative re-weighting algorithm to determine the optimal solution. If there is intensity corruption in multiple diffusion directions, the initial estimation of the tensor deviates substantially from the actual value, and robust fitting becomes unstable. Recently, Chang et al. [12] suggested an improved approach based on *a priori* knowledge that outlier data have lower intensity values. In this paper, we attempted a different approach, in which a non-fitting-based metric of data corruption was used as a part of the estimator. This metric was based on image intensity continuity through the slice orientation. In multi-slice imaging, adjacent slices are acquired at different time points, and, thus, the motion-caused corruption usually affects each slice independently, leading to the appearance of severe intensity discontinuity through the image slice orientation. An index, called the “corrected Inter-Slice Intensity Discontinuity” (corrected ISID, or cISID), was devised to reflect non-anatomical intensity discontinuity, and was tested for artifact detection. The performance of the corruption-detection accuracy was then compared to several types of robust estimators, with and without the cISID terms.

In addition to the examination of the corruption-detection accuracy, we also tested the feasibility of real-time image rejection and reacquisition methods. Corruption detection and data rejection are usually performed during post-processing tensor calculation. Although this is an effective approach, the rejection of corrupted pixels leads to a loss of SNR for the estimated tensor. To reduce these drawbacks of post-processing quality assurance, real-time monitoring of image quality, with reacquisition of severely corrupted images, is desirable. As a proof-of-principle study, we implemented a real-time monitoring and data reacquisition scheme on a scanner and the feasibility was evaluated.

## Materials and Methods

Retrospective case studies in this paper were conducted using existing clinical data. Approval was obtained from the Johns Hopkins Institutional Review Board to use these data after de-identification. All subjects participated in the real-time monitoring study provided written consent for participation in accordance under the oversight of the Johns Hopkins Institutional Review Board.

### Testing of different corruption detection methods

**Figure 1:**
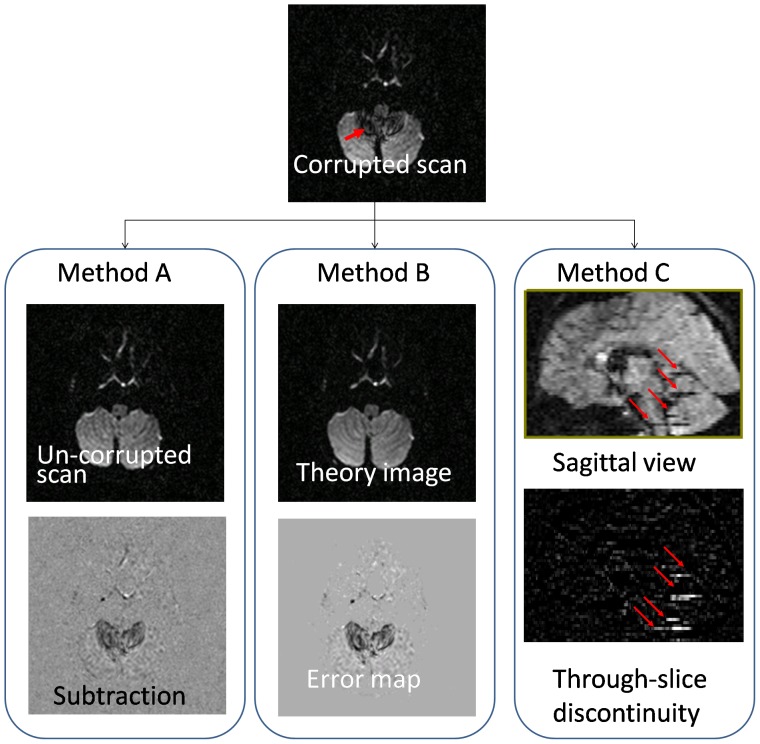
Three methods to detect motion-caused artifacts. Method A, subtraction of two repeated scans highlights artifacts; Method B, fitting residual (error) map shows artifacts as outliers; Method C, corrected inter-slice intensity discontinuity (cISID) of DW images is used to detect artifacts.

Corrupted pixels can be identified in three ways, as shown in Fig. 1. First, subtracted images from repeated DWIs can be used to detect artifacts, assuming that one of the repeated DWIs is not corrupted (Fig. 1, Method A). This approach can be used only when there are multiple repetitions and there are occasions when all repetitions happen to be corrupted. Second, tensor fitting errors could be used to detect corruptions, as shown in Fig. 1, Method B. Tensor-fitting-based artifact detection methods treat the outliers (points with large fitting errors) as corrupted points. Robust estimators that are relatively insensitive to the outliers have been proposed to detect artifacts, such as the Geman-McClure M-estimator (GMM) in Mangin et al. [11] and the Robust Estimation of Tensors by Outlier Rejection (RESTORE) in [10], [12]. In this study, a non-fitting-based criterion is introduced that is based on image intensity continuity through the slice selection orientation (cISID, Fig. 1, Method C). This method could be used individually or jointly with robust estimators to detect artifacts, which will be discussed in detail below.

### Least squares-based tensor fitting

According to the DTI theory, the relation between the b0 image (*S*_0_) and diffusion-weighted signals (*S* **_b_**) is as follows: *S* **_b_** = *S*_0_exp(−**b** ^T^ **Db**), where the vector, **b**, is decided by the diffusion gradients, and the diffusion tensor, **D**, is a 3×3 positive symmetric matrix (six independent variables) to be determined [13], and the superscript, T, represents vector or matrix transposition. S**_b_**s from at least six non-collinear **b**s are required to estimate the tensor. A widely used method estimates tensors by minimizing a least squares (LS) cost function:
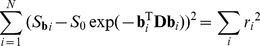
(1)the estimated tensor, 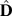 minimizes the cost function, which is the sum of the squares of the fitting errors (denoted by *r_i_*), *i* indicates the diffusion directions, and there are *N* DW signals in total. The fitted DW images calculated from 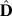 are called “theory images” as shown in Fig. 1, Method B, which is denoted as 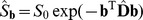. The image of fitting errors is called an “error map” in Fig. 1. (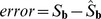). As the generic least squares method uses unconstrained tensor estimation, it could generate non-positive tensors during processing. To prevent that from occurring, positivity-preserving methods, which enforce the positivity of eigenvalues, can be used. A widely used method is the Log-Euclidean metric method [14], [15], where **D** is represented by its matrix logarithm **L** = Log(**D**), and the eigenvalues of **L** are the logarithms of the eigenvalues of **D**. No constraints on **L** are required during calculations, but the positivity of **D**, which is exp(**L**), is guaranteed. Thus the least square approach and its variations in this paper were adapted from the one proposed in [15] and were with respect to **L** instead of **D**.

### Robust estimators

In robust estimators, the cost function is a weighted sum of the error terms:
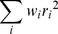
(2)


The definitions of the weighting, w_*i*_ (or noted as *w* *_robust_* for robust estimators), are different among these various methods. In [10], [11], the GMM was used, i.e., *w*_*i*_ = *w* *_robust_* = 1/(*r_i_* ^2^+*C^2^*), where *C* served as a normalization factor and was 1.4826 times the median absolute deviation (MAD) of the residue, *r_i_*, in [10]. The solution of the minimization problem is reached by an iterative reweighting method. It should be noted that robust fitting was used only for outlier detection. After robust fitting, the points with large errors were rejected as outliers and a non-robust fitting, using the unrejected points, was performed to arrive at the final tensor estimation.

In [16], the least trimmed squares (LTS) was proposed as a robust cost function:
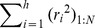
(3)where (*r_i_* ^2^)_1:*N*_ was the sorted square errors in ascending order, and *h* was a truncation factor, as the method attempts to minimize the sum of the first *h* smallest square errors.

Although appearing very different, LTS works as a robust estimator in (2) with special definitions of weighting, *w*_*i*_. In LTS, *w*_*i*_ are “0” at the terms with the largest *N*−*h r_i_* ^2^'s and “1” for the rest. LTS is similar in concept to GMM, as both give small weightings to large error terms and large weightings to smaller error terms, but LTS is more extreme, as it gives zero weightings to large error terms and equal weightings (one) to smaller error terms. However, the global solution of (3) is difficult to determine, since it requires an exhaustive search of all the 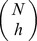 combinations of error terms. Alternatively, a method similar to the iterative reweighting was used in [16].

### ISID-based artifact detection

The pixel-by-pixel-based robust methods described above do not use any information outside the pixel of interest. In this study, we propose an image feature called Inter-Slice Intensity Discontinuity (ISID). The intuitive understanding of ISID is derived from the sagittal or coronal view of an image with artifacts, as shown in Fig. 1, Method C. In routine multi-slice imaging, slices are acquired in an interleaved spatial order, e.g., first the odd-numbered slices are acquired, then the even-numbered slices. In this method, adjacent slices are acquired at different time points, and, thus, the motion-caused corruption usually affects each slice independently, leading to the appearance of severe intensity discontinuity through the image slice selection orientation. The ISID can be detected by comparing the images before and after performing a morphology operation called “image close” [17] along the *Z* (slice) direction. It involves a dilation followed by an erosion operation, both in the Z direction. Considering a 3D image, *I*, as an array with subscripts (*i*, *j*, *k*)s, 0≤*i*≤*M*,0≤*j*≤*N*,0≤*k*≤*L*, the image after the close operation can be expressed as: *I*_2_ = close(*I*) = erosion(*I*_1_) = erosion(dilation(*I*)), such that for each (*i*, *j*, *k*), *I*_1_(*i*, *j*, *k*) = max(*I*(*i*, *j*, *k^+^*), *I*(*i*, *j*, *k* ^−^)), and *I*_2_(*i*, *j*, *k*) = min(*I*_1_(*i*, *j*, *k^+^*), *I*_1_(*i*, *j*, *k* ^−^)), where *k^+^* = min(*k*+1,*L*) and *k* ^−^ = max(*k*−1,0).

**Figure 2:**
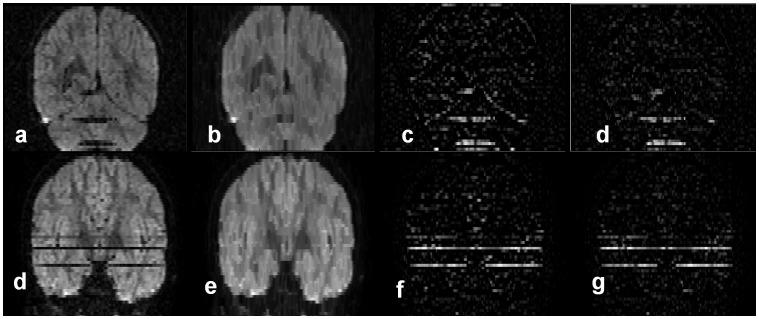
The process of the cISID calculation. **a**, original DW image in a coronal view; **b**, after the “close” operation along the slice selection (z) direction; **c**, the difference map between (**b**) and (**a**); and **d**, the final cISID map, in which the ISID of the average of all DW images was subtracted to suppress ISID due to through-slice anatomical changes. cISID calculation on another example with signal drop of several slices. (**e**–**h**): another example of whole slice intensity drop highlighted by cISID.

Intuitively, the close operation fills the ‘holes’ due to signal loss between slices. Fig. 2b is the result after the close operation performed on Fig. 2a. The difference between Fig. 2a and 2b is called the ISID image (Fig. 2c) and can highlight the artifact, although it is also contaminated by through-slice intensity changes due to the anatomy. To minimize the anatomical contribution, the ISID of a single DWI is then subtracted from an ISID image that is calculated from the average of all DWIs to obtain the corrected ISID (or cISID)(Fig. 2d). The image corruption could be regional, as shown in Fig. 2a, but, with severe head motion, the intensity of an entire slice could disappear, as shown in Fig. 2e, which could also be highlighted by cISID.

### Combination of robust fitting and cISID

The cISID itself can be used directly as a criterion for detecting corruptions, but it could fail if there happened to be artifacts at similar locations on multiple (>2) consecutive slices. However, because the cISID is based on information that is independent of the fitting errors, we hypothesized that the combination of these two criteria would enhance the stability of artifact detection. In robust estimators, the weightings are based on the assumption that *r_i_* terms with large errors should have small weightings. With cISID, the assumption is that the points with the large cISID values are more likely to be artifacts. To accomplish this, the weightings can be modified by multiplying a weighting derived from the cISID, which now becomes *w*_*i*_ = *w* *_isid_*·*w* *_robust_*, where *w*_*robust*_ is the robust weighting, as defined in GMM or LTS. The w*_isid_* is the weighting based on cISID. To reflect the effect of cISID, it should be a positive and decreasing function of |cISID| and have a maximum value at cISID = 0. This paper tested two forms of *w* *_isid_*: *w* *_isid_* = 1/(*cISID_i_* ^2^+*c* ^2^) and *w* *_isid_* = exp(−*cISID_i_* ^2^/*c* ^2^), where *cISID_i_* is computed from the *i*th diffusion direction image, and *c* is a normalization factor that is 1.4826 times the MAD of the cISID values of all the points inside the brain. If the *w* *_isid_* is multiplied by the *w* *_robust_* of GMM, it is noted as GMM-cISID, and if *w* *_isid_* is multiplied by the *w* *_robust_* of LTS, it is noted as LTS-cISID. In this paper, only the results of the first type of *w* *_isid_* (*w* *_isid_* = 1/(*cISID_i_* ^2^+*c* ^2^)) are presented because we did not find a noticeable difference among the two types.

In [15], another way to include the through-slice discontinuity is to use a spatial regularization term that penalizes tensor discontinuity between pixels/slices, and was added to the least squares cost function, as follows:
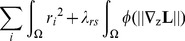
(5)where Ω is the image domain and all the tensors in the image are estimated simultaneously. **L** = Log(**D**) and 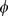 is increasing function [15] that penalizes large spatial variations of **L**. This method (noted as SR) was also compared with cISID-based methods. Here, we used only the gradient of **L** along the *Z* direction (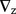), because, in the test, we wanted to penalize the large tensor changes between slices. *λ_rs_* is the weighting for the spatial regularization term.

We used iteratively re-weighted, nonlinear LS approaches to determine the solutions to all the weighted LS problems as in [10], [11]. Starting from an initial estimation of tensor obtained by a generic nonlinear LS fitting, in each iteration, the weighting terms were first calculated and the LS problem with fixed weighting terms was solved. For all the nonlinear LS fittings with fixed weightings and Log-Euclidean metrics in our experiments, a gradient descent method [15], with a step size decided by an adaptive line search, was used.

### Data Acquisition

Our experience indicates that image corruption occurs more in pediatric cases. In this study, existing clinical images of six pediatric patients were used. All patients were sedated during the scan and there was no detectable mis-registration of images due to bulk motion. The data were acquired using Avanto 1.5T and Trio 3.0T scanners (Siemens Medical Solutions, Erlangen, Germany), using a clinical, single-shot EPI sequence with a parallel imaging factor of two. The imaging matrix was 80×80 (FOV = 180×180 mm) or 96×96 (200×200 mm), and was then interpolated to twice the original matrix sizes. The slice thickness was 2–2.2 mm and 50–60 slices were acquired without gap. Diffusion weighting was applied along 12 independent orientations, with b = 800 s/mm^2^. One b0 image was also acquired. The scan took approximately two minutes, and was repeated two or three times. The repeated measurements were stored in separate files without signal averaging.

### Evaluation of the proposed methods

#### Pixel-by-pixel rejection for offline processing

Although we had at least two repeated datasets from each subject, only one set was used each time to test the automated outlier rejection methods. To identify corrupted pixels in images, subtracted images between two repeats (see Fig. 1, Method A) were thresholded (see details below) to generate a gold standard. This is not a perfect criterion because corruption could occur in both repetitions. Thus, an expert carefully inspected the two repeated images, and, manually defined the corrupted areas based on anatomical knowledge, in case both scans were corrupted In the datasets, 21 anatomical slices were used that contained at least one corrupted image among the 12 DWIs with different gradient encoding directions. These slices were grouped according to the number of corrupted DWIs. Among the 21 slices, there were six slice levels with one corrupted DWI, which were noted as group 1/12, eight slices with two corrupted DWIs (2/12), and seven slices with three or more corrupted DWIs (≥3/12).

Six methods were tested: GMM; GMM with cISID weighting terms (noted as GMM-cISID); LTS11; LTS11 with cISID weighting terms (noted as LTS11-cISID); adapted LS tensor estimation with spatial regularization of the Log-Euclidean of tensors (5), in which *λ_rs_* = 100. Their cost functions are summarized in Table 1. For the LTS approach, we tested the *h* values for 9–11, but only the results for *h* = 11 are shown because it performed slightly better than *h* = 9 and 10. Finally, a method with the cISID only (noted as cISID) was tested. The only differences between GMM and GMM-cISID or LTS11 and LTS11-cISID are in the definitions of the weightings; all other steps are the same.

**Table 1:** Cost functions and processing time for compared methods.

Method	Cost function	Processing times(s)
GMM	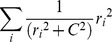	147
SR	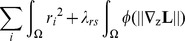	200
GMM-cISID	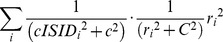	153
LTS*h*	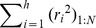	180 (*h* = 11)
LTS*h*-cISID	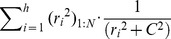	201(*h* = 11)
cISID		4

For each method compared, the following steps were performed:

1. For methods based on robust tensor estimators (GMM, SR, and GMM-cISID), a fitting error map was calculated by subtracting the raw DWI from the theory image. For cISID, the cISID image was used as the error map.
2. Error was normalized by estimated noise standard deviation, *σ*, using the method previously described [10], [18]. A threshold (*threshold_error_*) was applied to the normalized absolute fitting error map to create a binary (0/1 value) mask map (*mask_error_*), identifying pixels with normalized absolute errors above the *threshold_error_*.
3. The corrupted data points defined in the mask map were rejected from the final tensor calculation and the corrected tensor was calculated using the generic LS method.

All procedures were first developed in Matlab (MathWork Inc.) and then implemented in C++ language. The GMM and GMM-cISID were also implemented and are freely available in DTIStudio (www.mristudio.org) [19]. The software provides options to view the theory images, error maps, and the *mask_error_* to visually inspect the rejected pixels. The processing times (C++ programs) for different methods on a workstation with two 2.0 G processors, with 6 G RAM on a 160×160×52 dataset with 12 DWIs, are listed in Table 1.

### Comparison of the Performances by Receiver Operating Characteristic (ROC) analysis

The sensitivity/specificity of artifact detection was assessed as a function of the *threshold_error_*, and the results were compared against the gold standard. The gold standard images were also converted to binary *mask_error_* maps using a method similar to steps 2) and 3) described above, except that the error map was the difference between repeated scans, and with a fixed *threshold_error_* = 3 (three times the estimated standard deviation of the error map). The false-positive and false-negative pixels of the method under comparison were identified by simply subtracting the *mask_error_* map for that method from the *mask_error_* map of the gold standard. The resulting pixels with “0” values were considered “matched,” while “−1” indicated a “false positive” and “1” indicated a “false negative.” The false-negative (FNR) and false-positive (FPR) rates were calculated as a function of *threshold_error_*, and drawn as ROC curves.

For FPRs in the 0–50% range, area-under-the-curve (AUC) values of ROC from each slice of each tested method were calculated to assess performance. To compare the performances, first, a one-way analysis of variance (ANOVA) was performed on the AUC values from all methods to determine whether they had the same mean value. Then a Tukey's honestly significant difference (HSD) multiple comparison was done between each of the methods. In addition, paired *t*-tests were performed between GMM vs GMM-cISID and LTS11 vs LTS11-cISID to test the significance of the improvement.

### Comparison of the Performances by Comparing Tensor-Derived-Contrasts

To compare the impact of artifact detection accuracy on the tensor-derived contrast values after outlier rejection, the pixels in the tested slices were classified by the number of corrupted data points in the gold standard (1/12, 2/12, 3/12 and 4/12 data points corrupted). The mean diffusivity (MD) and fractional anisotropy (FA) values computed from the uncorrupted repeated scans were used as the gold standard. The MD and FA values computed from the corrupted repeats after outlier rejection using GMM and GMM-cISID were compared against the gold standard values. Paired *t*-tests were used to compare absolute errors between gold standard values and values from each of the GMM and GMM-cISID methods to determine which method achieved better estimation.

### Slice-by-slice rejection analysis

There are several important factors to be considered for real-time monitoring. First, the rejection must be done in a slice-by-slice manner, contrary to the pixel-by-pixel outlier rejection for the post-processing approaches. Second, an additional threshold (*threshold_area_*) for the number of corrupted pixels in each slice is needed to judge whether the slice should be reacquired, because it is not practical to reacquire the slices with a small number of corrupted pixels, which would lead to a longer scan time. For the slice-by-slice analysis, the gold standard was the rejection/non-rejection tag for each DWI slice. For each slice, the number of corrupted pixels was counted, based on the previous pixel-by-pixel gold standard, and the slice was classified as rejected when the corrupted area reached 1%, 1.5%, 2.0%, and 2.5% of FOV. Thus, four kinds of gold standards were generated, with a larger percentage of FOV representing a more conservative rejection strategy. For each type of gold standard, the ROC analysis was performed for three pixel-rejection methods: GMM; GMM-cISID; and cISID. For each method, a binary pixel-rejection mask was first generated for each slice with a fixed *threshold_error_* = 3. Then, the slice rejection was performed as a function of *threshold_area_*. By comparing these results against the gold standard, FNR and FPR were calculated and ROC curves were drawn. The gold standard is not available “on the fly” when performing online monitoring reacquisition. Thus, the performance of slice rejection algorithms can only be tested off-line. Seven single-repetition datasets, with all slices acquired from the pediatric subjects, were used for testing, and the AUC values calculated from each dataset were used for comparison. The same kind of statistical analysis as that used for the pixel-by-pixel rejection was then conducted to compare the performances of the three methods.

### Image reacquisition scheme

**Figure 3:**
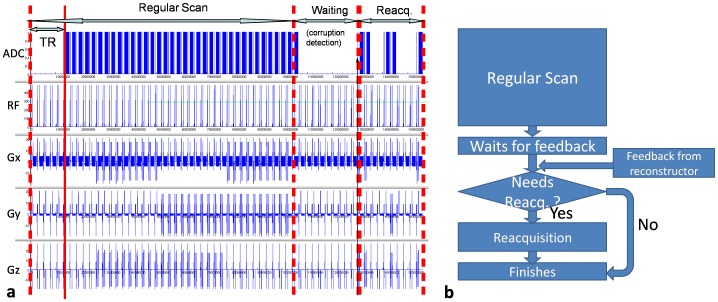
Image reacquisition. Pulse sequence timing scheme (**a**), and flow chart (**b**) of the image quality monitoring and reacquisition scheme. After the completion of a DTI scan, the quality of each slice is evaluated and those judged as corrupted are reacquired. The waiting time depends on the rejection algorithm and the data size (the dimensions of the images and the number of the gradient orientations).

As a proof-of-principle, the GMM-cISID method was implemented in the Siemens Image Calculation Environment (ICE) software package. ICE is a package used to develop programs that run on the scanner's reconstruction computer unit (reconstructer), including image reconstruction, online image processing, and the sending of feedback to the scanner controller to trigger reacquisition. The program was installed on a 3T Tim-Trio scanner (Siemens Medical Solutions, Erlangen, Germany). Images were processed online and rejected slices were reacquired after completion of the initial data set in real-time [20]. Fig. 3 shows a typical pulse sequence-timing scheme. After all the DWIs were received, the reconstructer began the artifact detection and slice rejection program using the method described in this paper. A message with “no rejection” was sent to the controller if no slice was rejected and the scan was finished. Otherwise, the information about the positions and diffusion directions of rejected slices was sent to the scanner controller. Then, the controller rescanned the rejected slices at corresponding positions and diffusion directions, and completed the scan. Upon receiving the reacquired slices, the reconstructer reconstructed them and replaced the rejected ones. During one TR, rejected slices with different gradient orientations can be acquired, unless the same slice level is affected by different gradient orientations. The sequence was tested on a human subject with a single-shot, diffusion-weighted EPI sequence with the following parameters: TR/TE = 6800/104 ms; matrix = 128×128 (FOV = 200×200 mm); 35 slices; and b value = 1000 s/mm^2^ in 20 directions. The *threshold_area_* = 150 pixels, which is about 1% of FOV.

## Results

### Comparison of different corruption detection schemes

**Figure 4:**
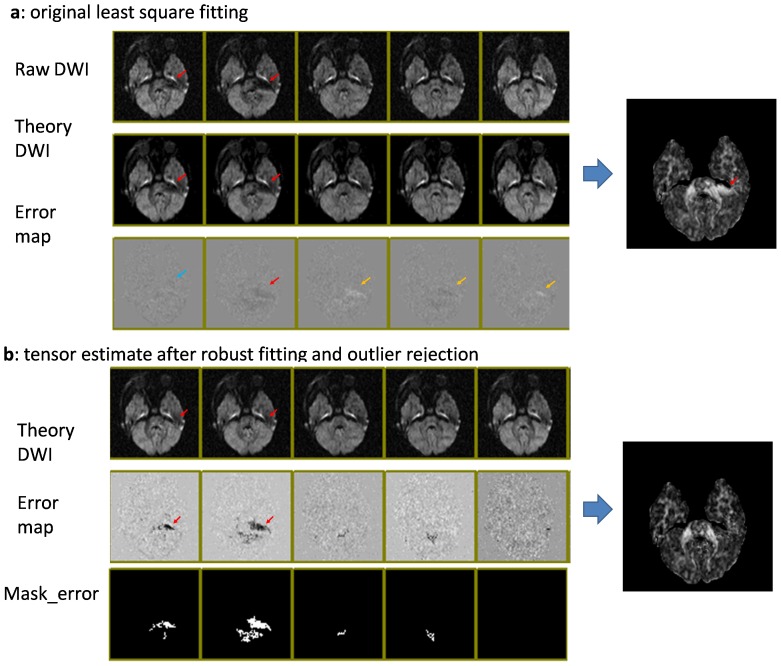
Comparison of tensor-fitting results, with and without outlier rejection. Red arrows indicate the locations of corrupted pixels. **a**: Results with conventional least squares fitting. The corrupted pixels cause faulty tensor fitting (theory images) and subsequent errors in FA values. Due to the fitting error, the error maps (fitting residuals) may not correctly highlight the corrupted pixels; not only corrupted pixels (blue arrows), but also some non-corrupted pixels (yellow arrows), reveal errors. **b**: Results with outlier rejection. If the pixel-rejection algorithm correctly rejects the corrupted pixels, the theory images are unaffected by the artifacts and the error maps accurately highlight the corrupted pixels. The images are screen shots from DtiStudio with which the post-processing rejection algorithms were implemented and tested.

Figure 4 demonstrates the effects of the outlier rejection. In the first row of Fig. 4a, the raw DWIs are shown, in which two of the 12 DWIs have apparent image corruption (indicated by the red arrows). Generic LS tensor fitting led to abnormally high FA values in the corrupted region. The theory images also indicate erroneous fitting results; because of the deviation of the fitting result, one of the corrupted DWIs has only minor errors (blue arrow), while the corruption-free DWIs have noticeable fitting errors (yellow arrows). Fig. 4b shows the results of tensor fitting with an outlier rejection. The final theory images (first row) are free from apparent artifacts, and the error maps could clearly define pixels with the artifacts. The resultant FA map did not have an abnormally high FA area.

**Figure 5:**
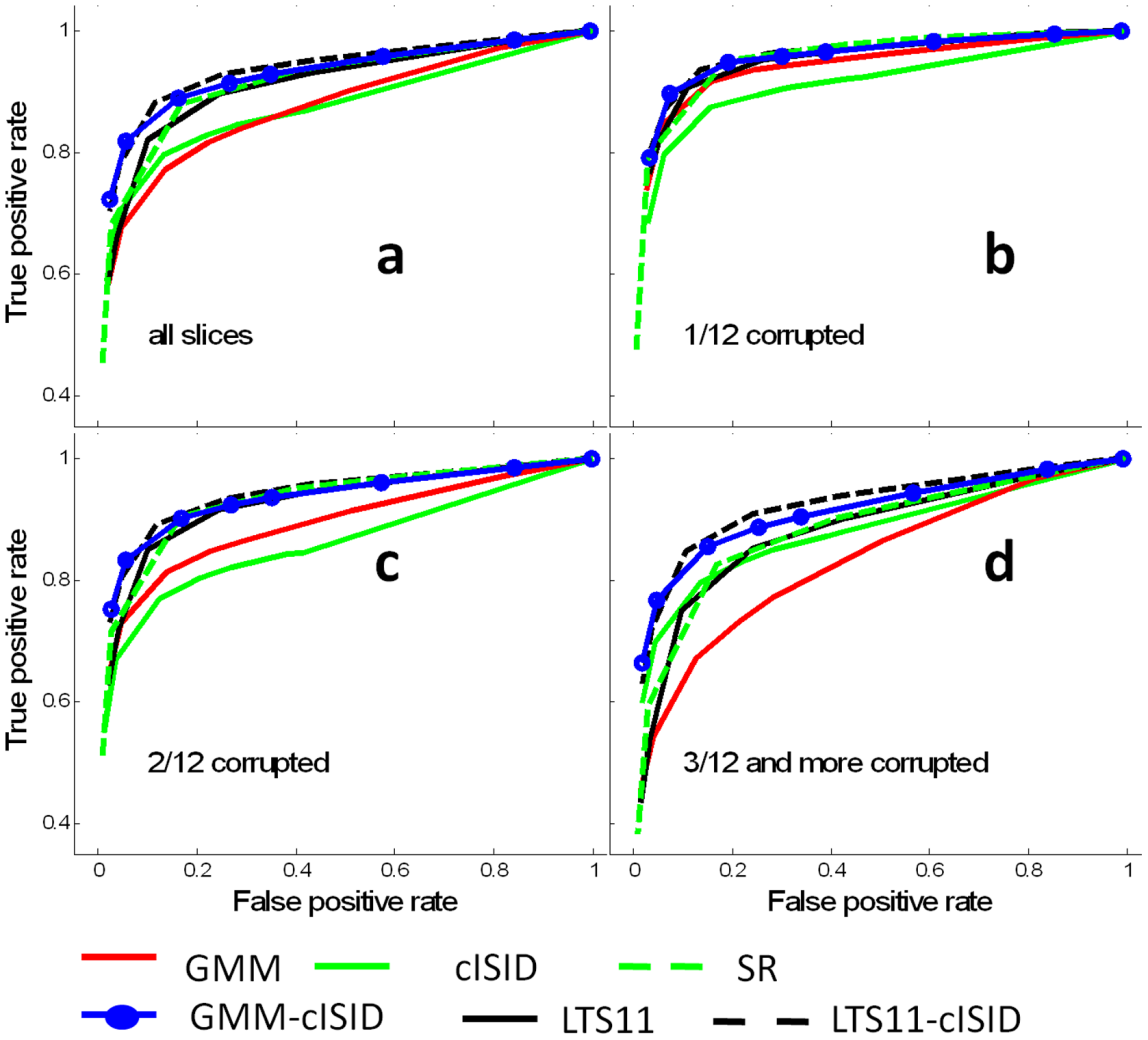
ROC curves of the pixel-by-pixel corruption detection methods. Six methods were compared, as indicated in the figure. Each ROC curve in the figures was calculated from the data of all the slices in the group. **a**: The results of all data combined; **b**–**d**: the ROCs with 1, 2, and ≥3 corrupted DWIs in the 12 diffusion orientations (1/12, 2/12 and ≥3/12).

Figure 5 shows the ROC analysis results of pixel-by-pixel rejections. The figure shows that the performance of the outlier rejection depends on the number of corrupted DWIs. Notably, spatially regularized (SR) tensor estimation demonstrates relative insensitivity to the number of corrupted data and performs better than GMM in the groups of 2/12 and ≥3/12 data. The use of the *w*_*isid*_ in GMM-cISID clearly stabilizes the performance of GMM and outperforms other methods. ANOVA tests showed that there were significant differences among the six methods (all *p*-values <0.01). The paired t-test showed significant improvement by adding the cISID term to GMM (*p*<0.01) and LTS11 (*p*<0.01) when there were more than two corrupted data points, which is also true for all slice combined (Fig. 5a, 5c and 5d). The Tukey's HSD showed that LST11, SR, GMM-cISID, and LST11-cISID perform consistently (*p*<0.05) better cISID and GMM results.

**Figure 6:**
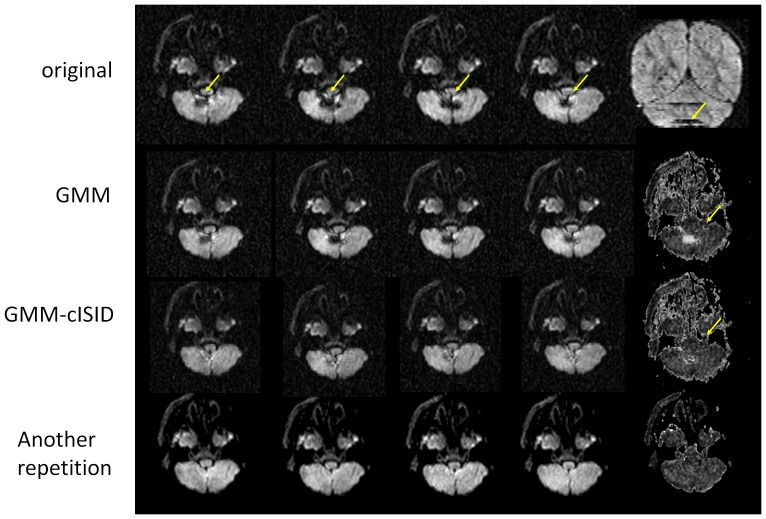
Demonstration of different rejection algorithms in a severely affected case. Top row: The four affected images in the axial views and one coronal view on the right (artifacts are demarcated by yellow arrows); 2^nd^ row: theory images (left four columns) and FA maps (right column) computed after outlier rejection using the GMM. 3^th^ row: GMM-cISID method with an FA map in the right column. 4^th^ row: another repetition.

Fig. 6 demonstrates actual data with four corrupted data points from the 12 orientation measurements in a cerebellum area adjacent to the fourth ventricle (indicated by arrows). GMM failed to reject the outlier pixels (demarcated by yellow arrows), resulting in erroneous FA values. The corrupted pixels had much smaller impacts on the GMM-cISID methods and the results were much more comparable to the other repetition.

**Figure 7:**
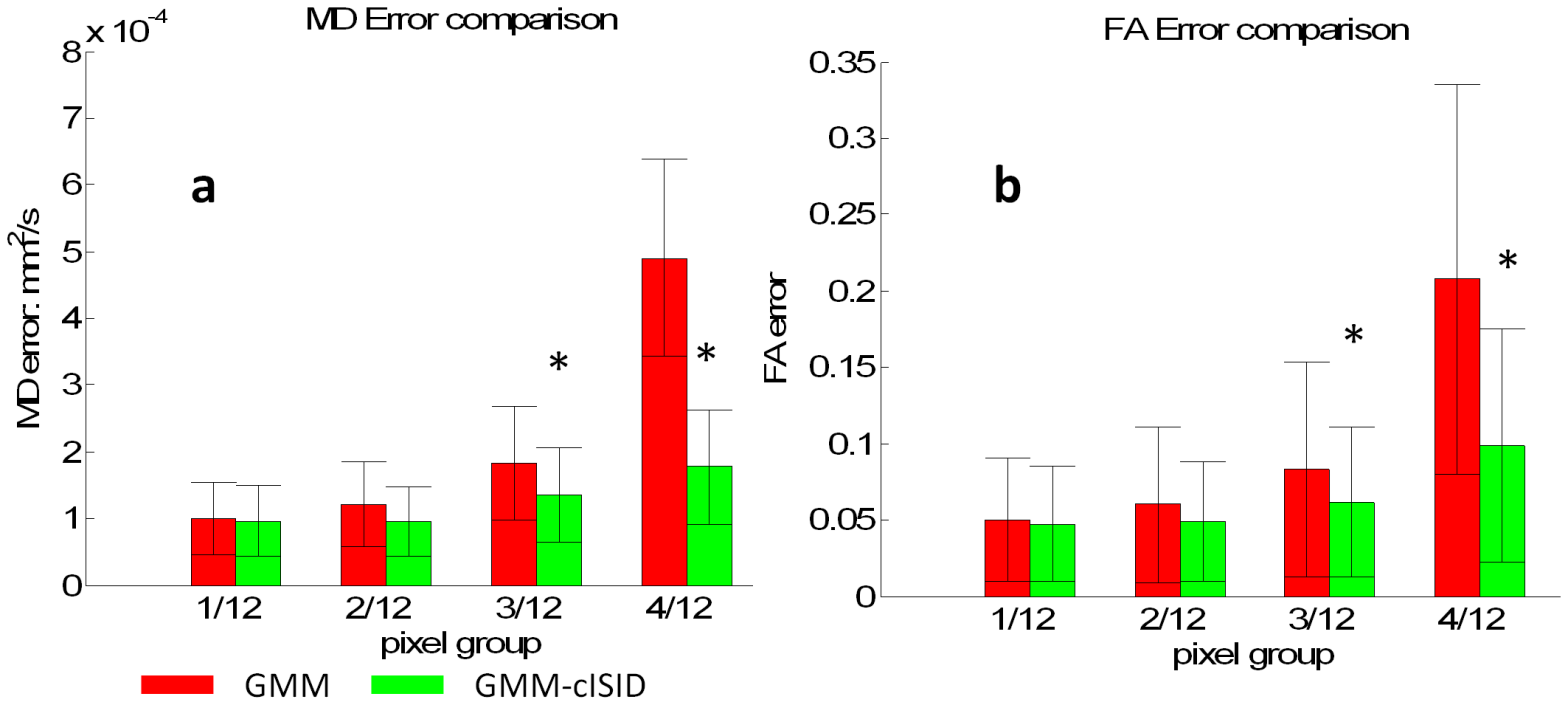
Comparison of estimation errors of tensor-derived contrasts by GMM and GMM-cISID against the gold standard. **a**: The results of MD values, with pixels grouped by the number of corrupted data points according to the gold standard. **b**: The results of FA values. Results with significant differences between two methods, as determined by *t*-tests (*p*<0.05), are marked by stars.

Fig. 7 shows quantitative comparisons of MD and FA values between automated rejection methods and the gold standard. The areas with corrupted pixels were defined the same way as the method used to define the gold standard in the ROC analysis above. The MD/FA values of these pixels were measured after outlier rejections by GMM and GMM-cISID. When only 1/12 or 2/12 data points were corrupted, both GMM and GMM-cISID methods had similar errors in MD and FA values, compared to the gold standard. When there were more than 3/12 corrupted data points, the GMM-cISID method had significantly smaller errors.

### Performance for slice-by-slice rejection

**Figure 8:**
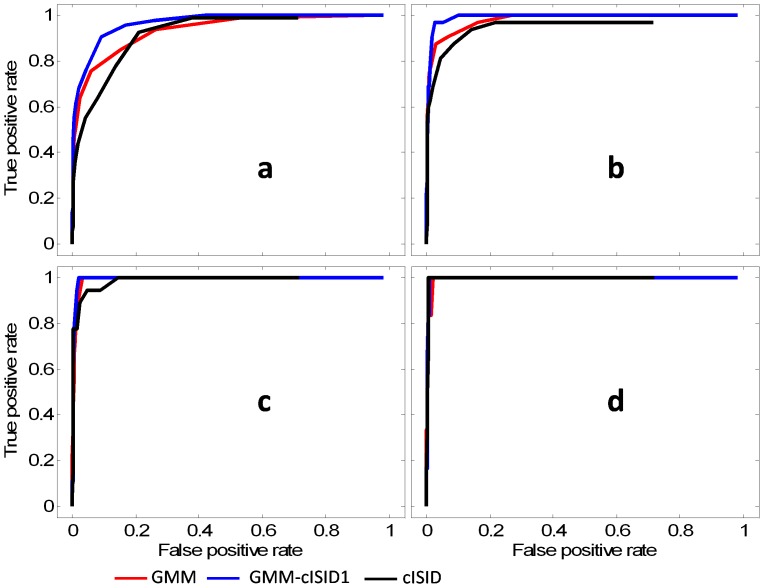
ROC curves of the slice-by-slice rejection methods; each curve in the figure was calculated from all the tested datasets combined. The slice rejection criterion of the gold standard was changed for 1% (**a**), 1.5% (**b**), 2.0% (**c**), and 2.5% (**d**) of the FOV, and the performances of GMM (red), GMM-cISID (blue), and cISID (black) were evaluated.

The ROC curves for slice-by-slice rejection are shown in Fig. 8. Fig. 8a–8d shows the results with different reject/no-reject gold standards: from the stringent (1% of FOV) to the conservative (2.5% of FOV) criteria for reacquisition decisions. Although the ROC curves show the superior performance of GMM-cISID and GMM to cISID, the Tukey's HSD does not show significant differences between the cISID and the other two methods, but paired *t*-tests showed significant differences between the cISID (AUC values: 0.467±0.038) and the GMM-cISID (0.481±0.017) method (0.477±0.020 for GMM) when the gold standard was most stringent (reject when 1% of the FOV was corrupted). When the rejection criterion reached 1.5% of the FOV (Fig. 8b), excellent accuracy was achieved with all three methods (all AUC values >0.49), and, at 2.5% FOV, all three methods reached almost perfect rejection performance (Fig. 8d).

### Testing of the real-time feedback and requisition scheme

**Figure 9:**
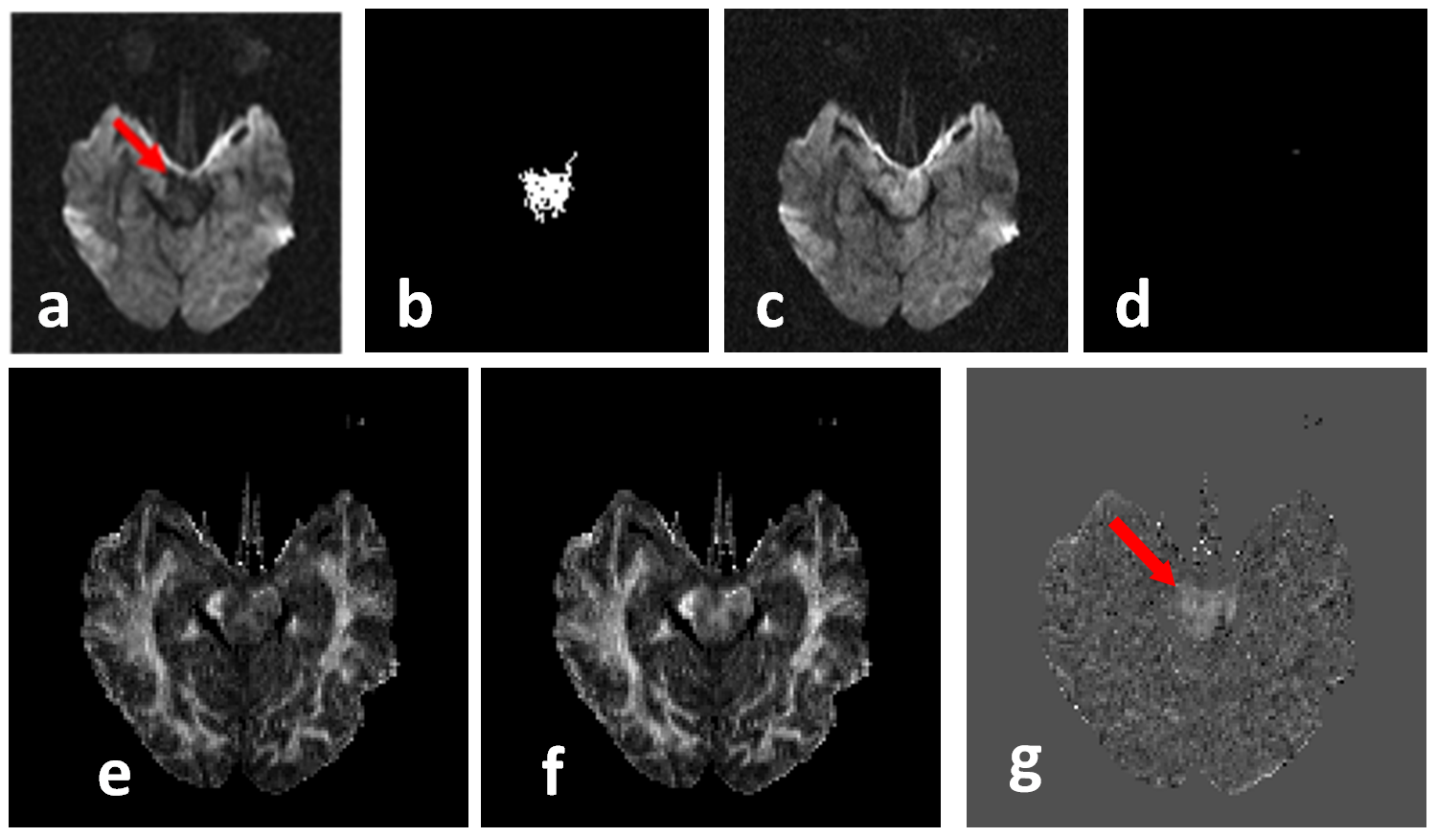
An example of a rejected image using the real-time image quality monitoring and reacquisition scheme. The image shown in (**a**) was judged as corrupted, based on the mask of error map (**b**), in which outlier pixels are indicated as white pixels. The reacquired image is shown in (**c**), which was free of corrupted pixels (**d**). The resultant FA map, with (**e**) and without (**f**) the rejection, led to a noticeable difference in FA (**g**).

The sequence ran successfully on a human subject [20]. The scan was completed in 2 m30 s, followed by 27 seconds of calculation (by GMM-cISID) time. The calculation of cISID only required one second with a 20-orientation scheme, 35 slices, and a 128×128 matrix size. Fourteen slices from five diffusion directions were rejected. The rejected slices were stored and the rejection accuracy was confirmed by agreement with the post-processing analysis. An example of rejected and reacquired images is shown in Fig. 9.

## Discussion

Motion-induced artifacts are often observed in DWIs, which could lead to significant inaccuracy in tensor calculation. Previous studies have suggested that one reason for the artifacts could be brain pulsation [6], [8], [21], [22], which could be effectively suppressed by cardiac gating [3], [5], [23]–[28]. However, the use of cardiac gating is often not a realistic solution because of the overly prolonged scan time. Consequently, cardiac gating is rarely employed in routine clinical DTI scans. The prevalence rate of such artifacts depends on the subject population. Our past experience demonstrates that pediatric cases tend to be more affected, especially at the areas around the 4th ventricle; it is not uncommon that 20% of DWIs are corrupted at such problematic brain regions (two to three corrupted DWIs in a 12-orientation scheme). Rejection of affected images or pixels could substantially improve the subsequent tensor estimation.

In this study, we evaluated the usefulness of the cISID (GMM-cISID) for fitting-based outlier rejection. When the number of corrupted data points was limited, the outlier rejection based on GMM and LTS11 was highly effective. The effect of the cISID term became apparent as the number of corrupted points increased, which clearly stabilized the rejection accuracy. We also demonstrated that the use of spatial regularization in tensor calculation, which takes into account the spatial continuity of tensor values, is also robust to the existence of artifacts. However, this method requires a much longer computation time than our GMM-cISID methods. The drawback of the cISID term is that if corrupted areas in two consecutive slices happen to overlap in the slice orientation, the detection sensitivity would be compromised. Therefore, for the post-processing analysis, we recommend that the cISID term be combined with a fitting-based estimator, as demonstrated in this paper.

Post-processing quality control, although it is effective, comes with a loss of SNR. This led to the idea of real-time quality monitoring and reacquisition of corrupted images. This approach, of course, also results in increased scan times. In this study, we implemented a feedback loop to monitor image quality and trigger reacquisition. The test was successful, and corrupted images were reacquired as expected. The current implementation, however, has several limitations. First, the fitting-based, pixel-by-pixel evaluation scheme requires a prolonged time for the calculation. The calculation time for the cISID term is negligible, and, thus, more suitable for real-time rejection criteria. The disadvantage of the cISID-based monitoring method is the level of detection accuracy, which is inferior to fitting-based algorithms. However, the performances of all three approaches tested in this study were excellent if the purpose is to detect severely affected images. The slice-by-slice rejection test (Fig. 8) shows the cISID had a performance similar to fitting-based criteria, in terms of slice rejection, when the size of the corrupted area for slice rejection was larger than 1.5% of the FOV, while all methods achieved almost perfect accuracy when the size of the corrupted area for slice rejection reached 2.5% of the FOV. There are several reasons that real-time monitoring should be used only for severe cases. First, the rejection of images with a small number of corrupted pixels would lead to severe lengthening of the scan time. Second, for cases with only a few corrupted pixels, we can expect excellent performance from a fitting-based detection method, such as GMM or the GMM-cISID methods for post-processing. Note that, in severe cases (with many corrupted pixels), the chances for multiple corrupted points for tensor calculation at a given pixel location would increase. Therefore, the role of real-time monitoring and reacquisition is to reduce the chances for multiple data corruption rather than to achieve near-perfect detection performance; thus, this method could play a complementary role with a post-processing, fitting-based pixel rejection.

In conclusion, a new criterion for artifact detection, cISID, was tested against several other detection methods. The methods using cISID significantly stabilized the performance of fitting-based outlier rejection for post-processing analysis when there were multiple corrupted points. A slice-by-slice rejection test showed cISID-based criteria accurately identified motion-corrupted slices when the size of the affected area was larger than 1.5% in a negligible computing time, compared with fitting-based methods, which make it suitable for online monitoring. Last, we tested the prototype of a real-time artifact detection and reacquisition method to enhance the image quality of DWIs. This study was based on only one DTI protocol with a fixed diffusion-weighting scheme, image resolution, and SNR. The independence of the cISID term on the diffusion tensor model could broaden its applicability to other types of diffusion approaches such as high-angular resolution diffusion measurements or potential non-tensor behavior in regions with a significant amount of crossing fibers [29]. Further testing under various imaging parameters, such as high b values (2000–3000 s/mm^2^) and other diffusion imaging methods, will be needed to establish a clinically stable and useful tool.
